# An Improved Artificial Bee Colony-Based Approach for Zoning Protected Ecological Areas

**DOI:** 10.1371/journal.pone.0137880

**Published:** 2015-09-22

**Authors:** Jing Shao, Lina Yang, Ling Peng, Tianhe Chi, Xiaomeng Wang

**Affiliations:** 1 Institute of Remote Sensing and Digital Earth, Chinese Academy of Sciences, Beijing, China; 2 University of Chinese Academy of Sciences, Beijing, China; Bangladesh University of Engineering and Technology, BANGLADESH

## Abstract

China is facing ecological and environmental challenges as its urban growth rate continues to rise, and zoning protected ecological areas is recognized as an effective response measure. Zoning inherently involves both site attributes and aggregation attributes, and the combination of mathematical models and heuristic algorithms have proven advantageous. In this article, an improved artificial bee colony (IABC)-based approach is proposed for zoning protected ecological areas at a regional scale. Three main improvements were made: the first is the use of multiple strategies to generate the initial bee population of a specific quality and diversity, the second is an exploitation search procedure to generate neighbor solutions combining “replace” and “alter” operations, and the third is a “swap” strategy to enable a local search for the iterative optimal solution. The IABC algorithm was verified using simulated data. Then it was applied to define an optimum scheme of protected ecological areas of Sanya (in the Hainan province of China), and a reasonable solution was obtained. Finally, a comparison experiment with other methods (agent-based land allocation model, ant colony optimization, and density slicing) was conducted and demonstrated that the IABC algorithm was more effective and efficient than the other methods. Through this study, we aimed to provide a scientifically sound, practical approach for zoning procedures.

## Introduction

China has been undergoing a period of economic reform and boom since the late 1970s, accompanied by rapid and widespread urbanization[1]. In 1978, 17.92% of China’s population lived in cities, but this percentage increased to 54.77% by 2014[2]. Land consumption is inevitably required to satisfy the growing population shift from rural to urban areas[3]. Farmlands, wetlands, and pristine mountains have been converted to urban land[4, 5]. This rampant expansion has led to serious ecological and environmental problems, including farmland erosion, climate change, species loss, water and air pollution, and soil degradation [1, 5–9]. Managing the tradeoffs between urbanization and ecological protection is a major challenge for Chinese policy makers.

China has altered its policy responses to meet this challenge by pledging to achieve socioeconomic and ecological sustainability in the urbanization process. A series of laws and regulations to protect specific resources have been issued since the mid-1990s, such as the Basic Farmland Protection Regulation, the Nature Reserve Regulation, and the Water Law. However, using farmland as an example, the amount of farmland has continued to decrease, particularly in expanding metropolitan areas and coastal regions [7, 10, 11]. Lately, to prevent the unlimited spread of urban areas and to protect important ecological land areas, a strict mandate named the Basic Ecological Control Line has been promulgated. According to the guidance in legislation, the national ascertained resources (such as nature reserves, basic farmland, water sources, and forest parks) must be embedded within the zoned protection, and a range of vegetation, water, and other land areas should also be incorporated to maintain the continuity and integrity of the regional ecological pattern. The zoning of protected ecological land areas has been implemented in fast-growing regions, such as Dongguan and Shenzhen [12–14]. Disorderly urban development has been effectively controlled [15, 16]. As the zoning is recognized as a reasonable measure to relieve land use conflicts, reduce environmental pressure, and ensure food security[17], it is underway in other cities in China. While brief guidelines to defining protected areas have been outlined above, they are difficult to follow in practice because of the difficulty in balancing conflicting interests of multiple officials and experts.

Zoning is a process of allocating different uses or activities to specific land units of area within a region [18, 19]. It inherently involves both site attributes (e.g., ecological benefit, human disturbance, and economic efficiency) and aggregation attributes (e.g., shape, contiguity, and compactness)[20]. The site attributes define the aptitude for certain uses. They are usually mapped based on GIS techniques, and these suitability maps are crucial for solving zoning problems. Generally, the assignment of each land unit to a specific use will partly or completely depend on the agreement between the site attributes [17]. Aggregation attributes are vulnerable to being ignored, but their importance for ecological protection has been widely discussed in the literature[21]. Reducing the fragmentation of spatial patterns may increase agricultural productivity and successful species dispersal, mitigate human impacts, improve rural scenic quality, and facilitate reserve management [22–26].

Scientific interest in the zoning of protected natural areas has been present since the creation of the first national park in 1872 [17]. Carver (1991) proposed a hierarchical optimization method to first allocate the most suitable areas for the highest priority use and then assign the areas for the second-highest priority use; this process continues until the entire surface area is allocated[27]. Barredo (1996) proposed the ideal point analysis method for conflicting land uses, which aims to maximize the suitability for one land use while minimizing the suitabilities for the remaining uses[28]. Li and Yeh (2001) introduced a Density Slicing (DS) method to zone the best land for agricultural protection, in which the NDVI image that reflected the quality of agricultural land was sliced by a threshold value, and the slicing threshold was decided by including a sufficient number of pixels that could satisfy the quota of zoning [23]. These methods are implemented under a raster environment and are based entirely on the suitability values without aggregation constraints, which will result in the fragmentation of land-use patterns. Liu and Li (2008) embedded some adjustment operations after designing the initial core zone based on habitat suitability assessment; they deleted the small patches with a threshold value, and merged the remaining big patches together with those non-core zone patches which surrounded by these big patches, then obtained the final core zone which was aggregate and organized [29]. This kind of adjustment operations is common in practice and conducted mostly through the use of thresholds based on area, shape and connectivity indices to remove and group grid cells. The adjustment is qualitative and rely heavily on the experience of the operators [17, 22]. To overcome this problem, Geneletti and Van (2008) regarded the homogeneous land units instead of the single grid cells as the zoning spatial elements, and then selected units based on their suitability rank; they considered the zoning scheme to be a directly implemented one[30]. The method with gathered cells can produce a relatively compact pattern to some extent; however, the challenge of handling complex aggregation constraints remains.

Researchers have successfully formulated mathematical models to incorporate aggregation attributes as objective functions as well as incorporate site attributes[31]. Thus, zoning becomes a combinatorial optimization problem of simultaneously optimizing features related to the allocation process, such as ecological benefit and spatial morphology[15]. The mathematical method has been proposed as an effective way to quantitatively zone and reasonably handle spatial constraints. However, optimal patch design is a difficult geometric problem [32]; zoning protected ecological areas under spatial constraints belongs to the NP-hard problem with a huge complex search space. Meanwhile, the search space has increased exponentially with the growth of both the study area and the spatial resolution, making the problem of zoning protected ecological areas even more challenging. Effective optimization methods should be adopted because exact enumeration methods cannot solve such difficult problems in a reasonable amount of time. Many efforts have been made to solve the mathematical model. Initially, mathematical techniques, such as linear programming (LP) and mixed integer programming (MIP), were often used. Cocks and Baird (1989) applied LP to solve multiple reserve selection problems in South Australia[33]. Hof and Joyce (1993) developed an MIP approach for spatially optimizing wildlife and timber in managed forest ecosystems[34]. Aerts et al. (2003) used LP to solve multi-site land-use allocation, and they noted that although optimal solutions could be found by these techniques, the computation time was far beyond the acceptable scope when the study area was larger than 30 x 30 cells[31]. Later, meta-heuristic algorithms were proven to be fast and to have operating advantages for solving complex combinatorial optimization problems. Scholars applied traditional artificial intelligence methods, such as the simulated annealing (SA) and genetic algorithm (GA), to land-use planning with some success. For example, Aerts and Heuvelink (2002) proposed a land-use model to maximize developing consumption and minimize spatial compactness, which they solved using SA[35]. Verdiell et al. (2005) developed an SA method for the zoning of protected natural areas subject to both box and spatial constraints[17]. Santé-Riveira et al. (2008) proposed an expanded SA method for land allocation[36]. Stewart et al. (2004) applied the GA to multi-objective land-use planning[18]. However, most of these methods were applied to spatial data with coarse resolution and would provide no improvements in solution quality or computation time with increased study area size or spatial resolution[18, 37].

Recently, multi-agent methods and emerging artificial intelligence methods were employed to solve protected area zoning and land-use allocation problems in large areas. For example, Chen et al. (2010) proposed an agent-based land allocation model (AgentLA) with contiguous constraints, which they applied to zoning for natural conservation[15]. Liu et al. (2011) revealed an integrated approach of remote sensing, GIS and the ant colony algorithm (ACO) for zoning protected ecological areas[5]. Li et al. (2011) developed a method combining cellular automata and the ACO to zone natural areas in a changing landscape[16]. Liu et al. (2011) proposed an approach for farmland zoning that was based on an artificial immune system[10]. Liu et al. (2013) considered social and economic factors and spatial conditions; they developed a method based on particle swarm optimization and system dynamics for land allocation[38].

Artificial bee colony (ABC), a new heuristic bionic algorithm inspired by the foraging behavior of bees, was first proposed by Karaboga (2005) for multi-variable and multi-modal continuous function optimization[39]. Previous research on the ABC mainly focused on comparing its computing performance with other heuristic algorithms, and the ABC was found to be capable of finding an optimal solution with greater probability than other algorithms[40, 41]. The algorithm was then used to solve combinatorial optimization problems and discrete domain problems, such as training neural networks[42], wireless sensor networks[43], and discovering transition rules for cellular automata[44], and it has produced good experimental results. Studies have shown that the ABC is able to escape local optima, avoid premature convergence, and maintain the characteristics of adaptive, diversified, dynamic learning with distributed computation and memory function, thereby making it possible to solve complex spatial optimization problems.

Establishing ecological protected areas is an important measure for the Chinese government to protect limited ecological resources during urbanization. There is an urgent need for a reliable, flexible, and efficient approach that can provide a rational and transparent overview of the consequences of incorporating different perspectives from officials and experts at the stage in which formal consultations for zoning ecological protected area have yet to be undertaken in some cities. Through the present study, we explore an ABC-based approach for protected area zoning at a regional scale and evaluate whether it can quantitatively identify the optimal scheme under specific objectives, constraints and data. To our knowledge, this is the first study on zoning protected ecological areas using an ABC algorithm. The mathematical model is formulated to generate protection patterns that maximize ecological suitability and spatial compactness while minimizing urban development potential. The algorithm is first verified with simulated data and then used to generate an optimal scheme of ecological protection areas in Sanya, a rapidly developing ecotourism city in China, as an applied example. Finally, a comparison with existing algorithms is conducted.

## Model and Data

### Model

In this study, the following three objectives were adopted: (1) maximize the ecological suitability, (2) minimize the urban development potential, and (3) maximize the spatial compactness. The first two objectives originate from site attributes. The ecologically protected area should occupy the area with high ecological benefit for the initial establishment purposes. The potential of urban development should be incorporated because of two considerations: one is that economic boom should not be totally hindered by ecological conservation, and the other is that an ecologically protected area near urban land may be subject to stress from adjacent human activity. Therefore, we regard the urban development potential as a negative effect. The last objective is an aggregation attribute, and its importance is stated in the preamble. These objectives conflict with each other to some extent and have been widely used in the literatures [5, 10, 15, 16, 29]. Mathematically, the objectives are formulated as follows:

$$\text{Maximize}\;\text{Ecological}=\sum_{\text{i}}{\text{Eco}}_{\text{i}}{\text{x}}_{\text{i}}/\text{Q}$$ (1)

$$\text{Minimize}\;\text{Development}=\sum_{\text{i}}{\text{Dev}}_{\text{i}}{\text{x}}_{\text{i}}/\text{Q}$$ (2)

$$\text{Maximize}\;\text{Compactness}=\sum_{\text{i}}{\text{Com}}_{\text{i}}{\text{x}}_{\text{i}}/\text{Q}$$ (3)

$${\text{Com}}_{\text{i}}=\sum_{\text{n}\times \text{n}}{\text{x}}_{\text{i}}/\sum_{\text{n}\times \text{n}}{\text{D}}_{\text{i}}$$ (4)

In these models, Eco_i_, Dev_i_, and Com_i_ denote the ecological suitability, development potential, and spatial compactness, respectively, of land unit/cell i (the land unit denotes the spatial element for zoning and can constitute a single grid cell or a set of cells; here, we use a grid cell as the land unit). The value x_i_ indicates whether the cell i is protected. If it is selected for protection, x_i_ = 1; otherwise, x_i_ = 0, where ∑_i_x_i_ = Q and Q is the quantity of protected ecological area in the cells. Compactness is evaluated by neighborhood density, which is the proportion of ecologically protected cells among the configurable cells in the neighborhood. D_i_ indicates whether i is a configurable unit, and n donates the setting size of the neighborhood.

This task is a multi-objective problem, which is generally solved by a simple linear weighted method [16, 45]. Thus, the model for zoning a protected ecological area can be identified as follows:

$${\text{Maximize}\;\text{w}}_{1}\times \text{Ecological}+{\text{w}}_{2}\times \left(1-\text{Development}\right)+{\text{w}}_{3}\times \text{Compactness}$$ (5)

where w is the weight for each sub-object and is subject to w_1_ + w_2_ + w_3_ = 1.

### Study area

The study area is Sanya, an ecotourism city located in the southernmost of Hainan Island, China, beside the South Sea. The northern portion of the city is occupied by mountains and forest, whereas the southern and eastern portions constitute a coastal zone with low-lying and fertile land suitable for human habitation and travel. In recent years, the urban land of Sanya has greatly expanded due to economic development and tourism-based prosperity, resulting in a large number of environmental and ecological problems. City officials have realized the need to distinguish ecologically controlled regions from urban development regions, and an urban overall planning for the year 2020 has been issued, asserting to plan 1,533.1 km^2^ for ecological protection, representing 79.9% of the entire city.

**Fig 1:**
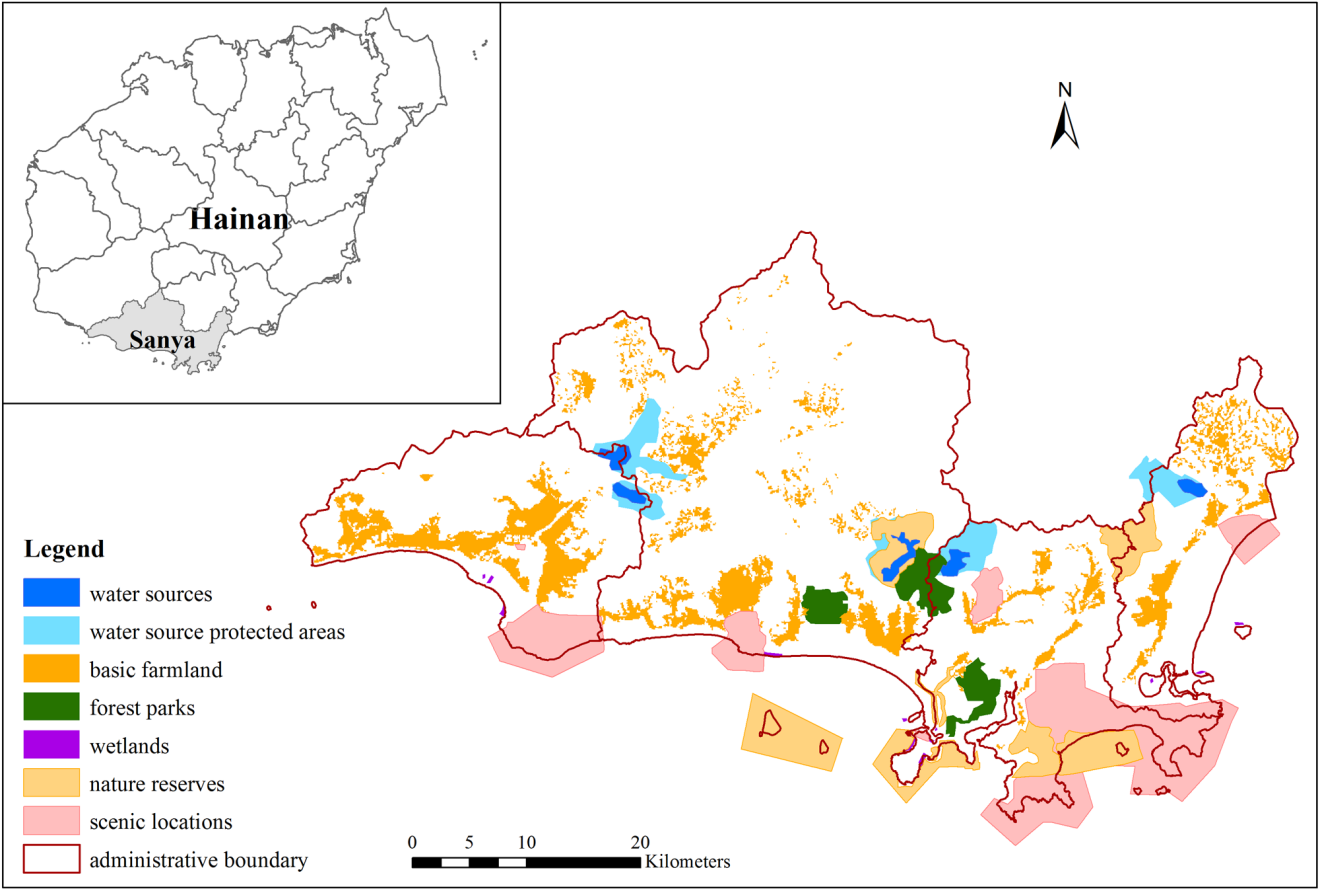
Location of Sanya and its national ascertained resources.

According to the guidance for zoning ecologically protected areas in the legislation described in introduction, national ascertained resources are necessary elements in the protected zone. In Sanya, these ascertained resources include water sources and their protected areas, basic farmland, forest parks, wetlands, natural reserves, and scenic locations (details in Fig 1, which are digitized from the urban overall planning of Sanya). In addition, a range of vegetation, water, and some other lands that are not included in the above resources should be incorporated to maintain the continuity and integrity of ecological pattern; this additional amount comprises 758.1 km^2^ and is the core in the planning.

### Data and processing

A basic component of the planning process involves obtaining evaluated maps for the objectives corresponding to site attributes, which represent the degree of achievement of goal requirements on each land unit [46]. The evaluation always involves a number of spatial variables / factors. Generally, it can be handled by integrating the use of GIS and multi-criteria decision analysis (MCDA)[47–49]: first, raster-based coverage for each evaluation factor is obtained by GIS technique, and then MCDA is used to group these factors into a comprehensive map which can serve as the basis for subsequent planning[22].

In this study, the ecological suitability map and urban development potential map should be generated according to the model. Thus, two spatial multi-criteria evaluations were performed, and a common weighted linear summation method was adopted, where the weight for each spatial variable was calculated by the analytical hierarchy process[50]. Table 1 and Table 2 show the criterion trees and weights for evaluating ecological suitability and urban development potential. Ecological suitability was evaluated based on vegetation, terrain, water, soil, and land-use, and the top criteria for urban development potential were urban aggregation, scenery attraction, and traffic superiority. Criteria selection and weight setting relied as much as possible on consultations with experts and review of the literatures [5, 15, 16], and the assessment was based on representative, reliable and available data.

**Table 1:** Criterion tree and weight for evaluating ecological suitability.

Criterion (Weight)				Label
Vegetation	0.41620	Fractional vegetation cover	0.41620	a
Terrain	0.09860	Slope	0.02360	b
		Elevation	0.01350	c
		Slope orientation suitability	0.06140	d
Water	0.16110	Distance to water	0.16110	e
Soil	0.06240	Soil texture suitability	0.06240	f
Land use	0.26180	Land use suitability	0.26180	g

**Table 2:** Criterion tree and weight for evaluating urban development potential.

Criterion (Weight)				Label
Urban aggregation 0.64790		Density of urban area	0.41980	h
		Distance to city center	0.14890	i
		Distance to district centers	0.07920	j
Scenery attraction 0.12220		Distance to coastline	0.04070	k
		Distance to scenic locations	0.08150	l
Traffic superiority 0.22990	Road density 0.05510	Density of road network	0.05510	m
	Distance to transportation hubs 0.03160	Distance to airports	0.00380	n
		Distance to railway stations	0.01420	o
		Distance to bus stations	0.00820	p
		Distance to expressway entrances	0.00540	q
	Distance to transportation lines 0.14330	Distance to railways	0.01520	r
		Distance to expressways	0.03730	s
		Distance to main roads	0.09070	t

**Fig 2:**
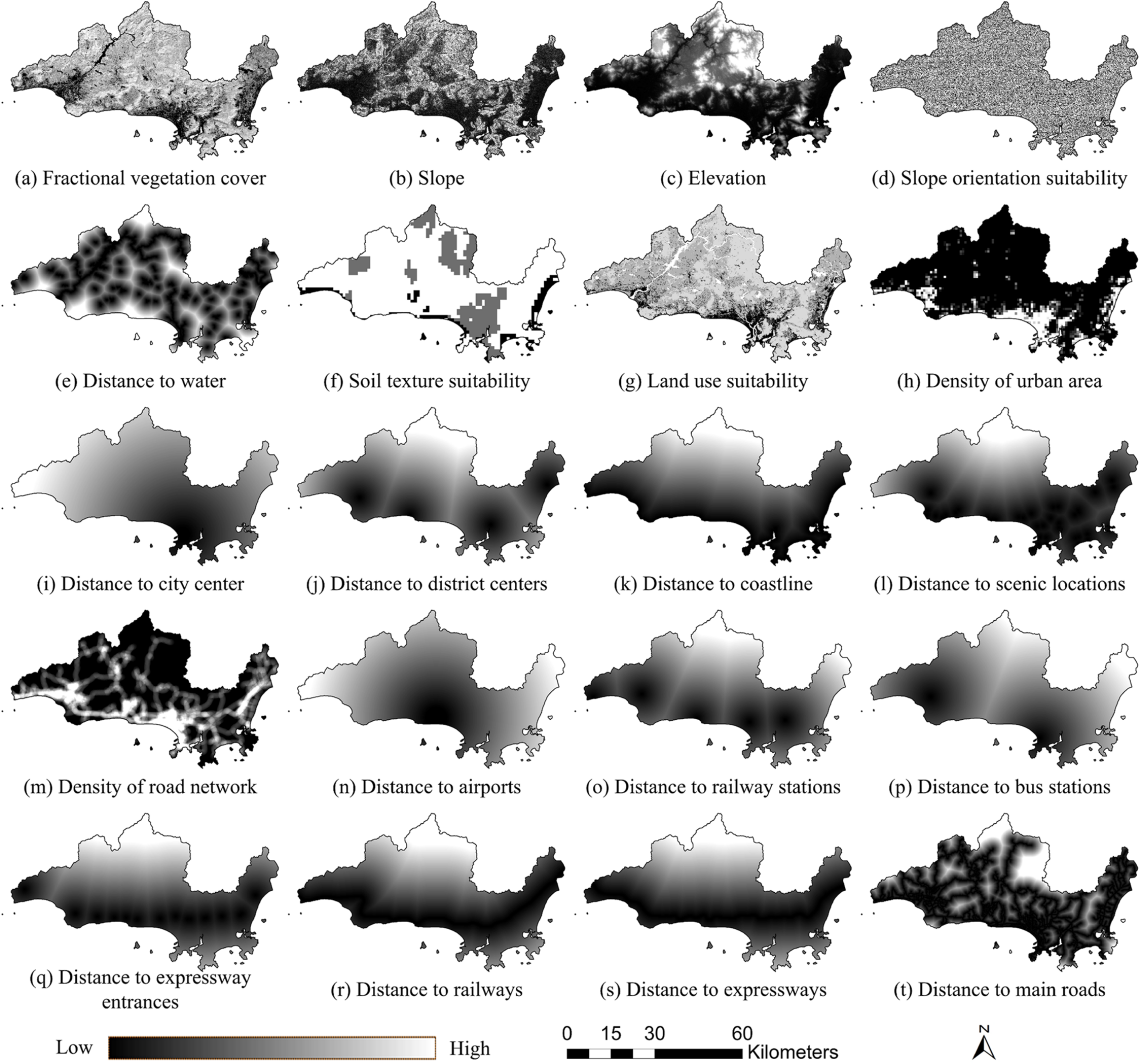
Various spatial variables for multi-criteria evaluations.

**Fig 3:**
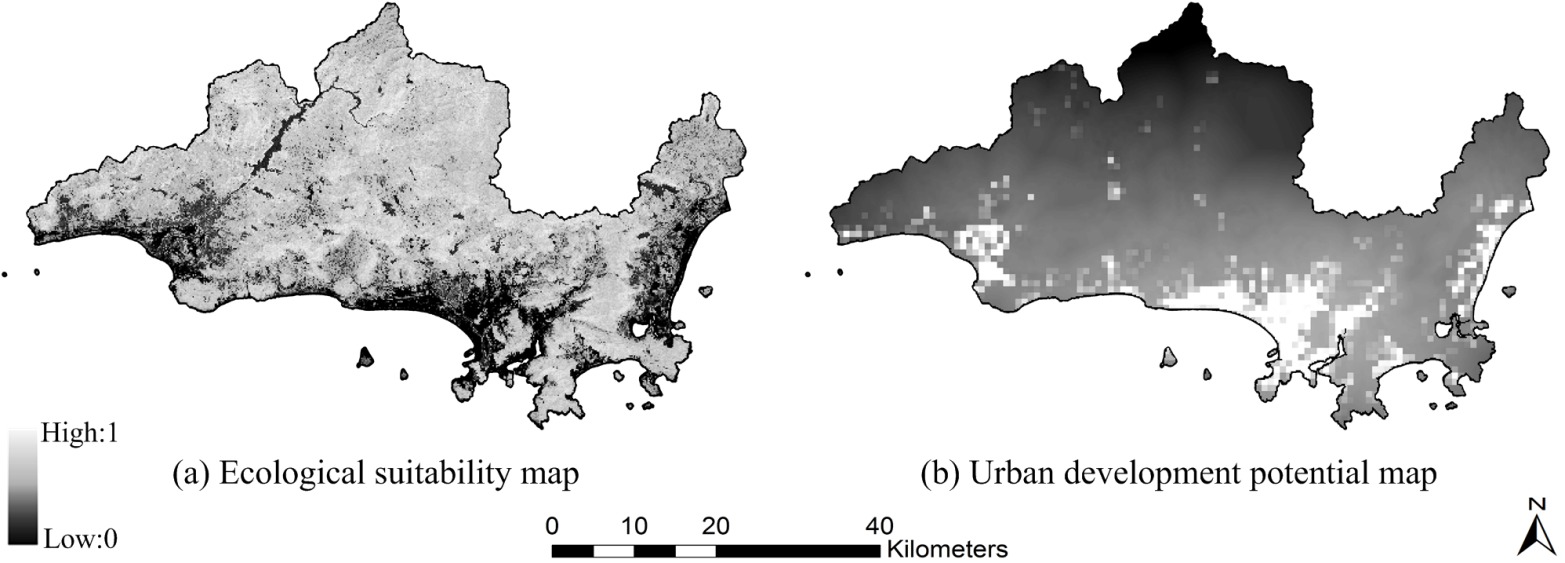
Ecological suitability and urban development potential maps of Sanya. (a) Ecological suitability map. (b) Urban development potential map.

We acquired a Landsat-8 image of Sanya (captured on October 26, 2013, http://glovis.usgs.gov/), DEM data of ASTER (http://gdem.ersdac.jspacesystems.or.jp/), soil texture data from the Second Nationwide Land Survey (http://westdc.westgis.ac.cn/data), and basic geographic data for the city. Twenty spatial variables evaluating ecological suitability and urban development potential were extracted and transformed to normalized raster data consisting of 603×345 cells with a spatial resolution of 150 m using ArcGIS software (details in Fig 2 and S1 Data). Fig 3 shows the generated ecological suitability map and the urban development potential map, which are both comprised of continuous numerical values.

Expert knowledge from ecologists and managers as well as the authors of the present study made it possible to define the evaluation scheme. As stakeholders and experts can have different views on the relative importance of the criteria, it might be difficult to integrate these opinions together. To test the methodology, we established a set of weights that have the majority agreement from the group of experts that participated in the research. However, different opinions should also be considered in practice; for this reason, the approach detailed here enables multiple runs using different weight sets corresponding to different viewpoints. Moreover, the selection of the above spatial variables is subject to data availability; if other useful variables are available in the future, they can be included in the evaluation procedure, with the applicability of the proposed method remaining unchanged.

## Methodology

The basic ABC algorithm was originally designed for continuous function optimization[39]. To make the algorithm applicable to zone ecologically protected areas and to solve the problem effectively, a novel discrete version of the ABC algorithm, named the improved artificial bee colony (IABC) algorithm, is proposed in this section. The approach includes a combined strategy to initialize the bee population, a “replace-and-alter” strategy to generate neighbor solutions, and a “swap” strategy for local search. A detailed description of the algorithm follows.

### Basic ABC

In the basic ABC algorithm, high-quality nectar sources can be found via communication among three groups of foraging artificial bees, namely, employed bees, onlookers, and scouts. The algorithm begins with a number of food sources (candidate solutions for optimization problems) that are randomly generated. Then, the following three steps are repeated until a termination criterion is met[41]. First, the employed bees are sent to each food source and the amounts of nectar are then measured (evaluated by fitness of solutions), with the highest-quality food source retained using the greedy selection mechanism. Second, the food sources are select by the onlookers after the employed bees share information according to the roulette wheel mechanism, and the retained food sources are determined. Third, if a food source is not updated within a limited number of repetitions, a scout is sent out to generate a new possible food source randomly.

Three control parameters should be set in the basic ABC algorithm: (1) SN: the number of food sources; (2) Limit: the number of repetition cycles to activate a scout bee. If a food source cannot be improved further in “limit” cycles, then it will be abandoned and replaced by a new food source generated by scout bee; this is a particular phase of bee-based algorithm to skip out of local optimum[40, 51]; and (3) MCN: the number of maximum cycle iterations, which is a termination criterion.

The probability of each food source chosen by the onlookers is as follows:

$${\text{p}}_{\text{j}}=\frac{{\text{fit}}_{\text{j}}}{\sum_{\text{n}=1}^{\text{SN}}{\text{fit}}_{\text{n}}}$$ (6)

where fit_j_ denotes the fitness of solution j. The larger fit_j_ is, the more likely solution j is to be chosen. The model in this study is a maximization problem, meaning that the goal is to find the maximum of object function value in formula (5). Therefore, the fitness of a feasible solution is equal to its object function value.

### Solution representation

**Fig 4:**
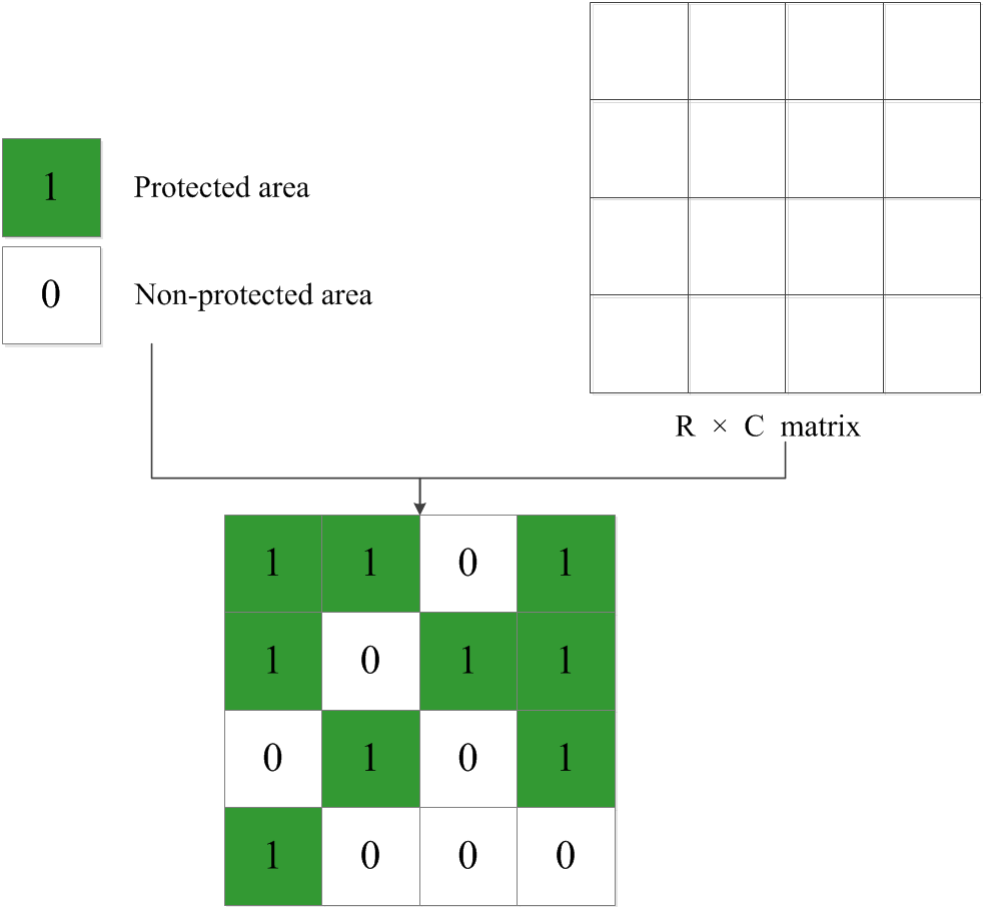
Representation of a solution for zoning protected ecological area.

In the problem of zoning of protected ecological areas, a candidate solution can be represented by a binary array that is as large as the study area. A cell allocated for protection will be encoded by 1 and is otherwise encoded by 0, as shown in Fig 4.

### Population initialization

To guarantee an initial population with a specified quality and diversity simultaneously, two different methods were utilized together to generate initial solutions as the food sources. (1) The “complete-random” method randomly selects cells from candidate ones in the study area and configures them as protected areas. This is the traditional method adopted by many algorithms in the literatures [5, 15, 17]. (2) The “pseudo-random” method is the second method used. The model consists of site objectives (ecological suitability and urban development potential) and an aggregation objective (compactness). The aggregation objective value of each land unit cannot be attained until a spatial pattern has been allocated, whereas the site objectives of each land unit are preconditioned in data processing procedure and can thus be regarded as guidance for allocation. Therefore, we placed site attributes in a prominent position and developed a novel initial method. First, a specified number of cells with the best site attribute value are allocated. Second, remainder cells are selected through roulette, guided by the ratio of the site attribute value of each cell to the summation of all configurable cells.

**Fig 5:**
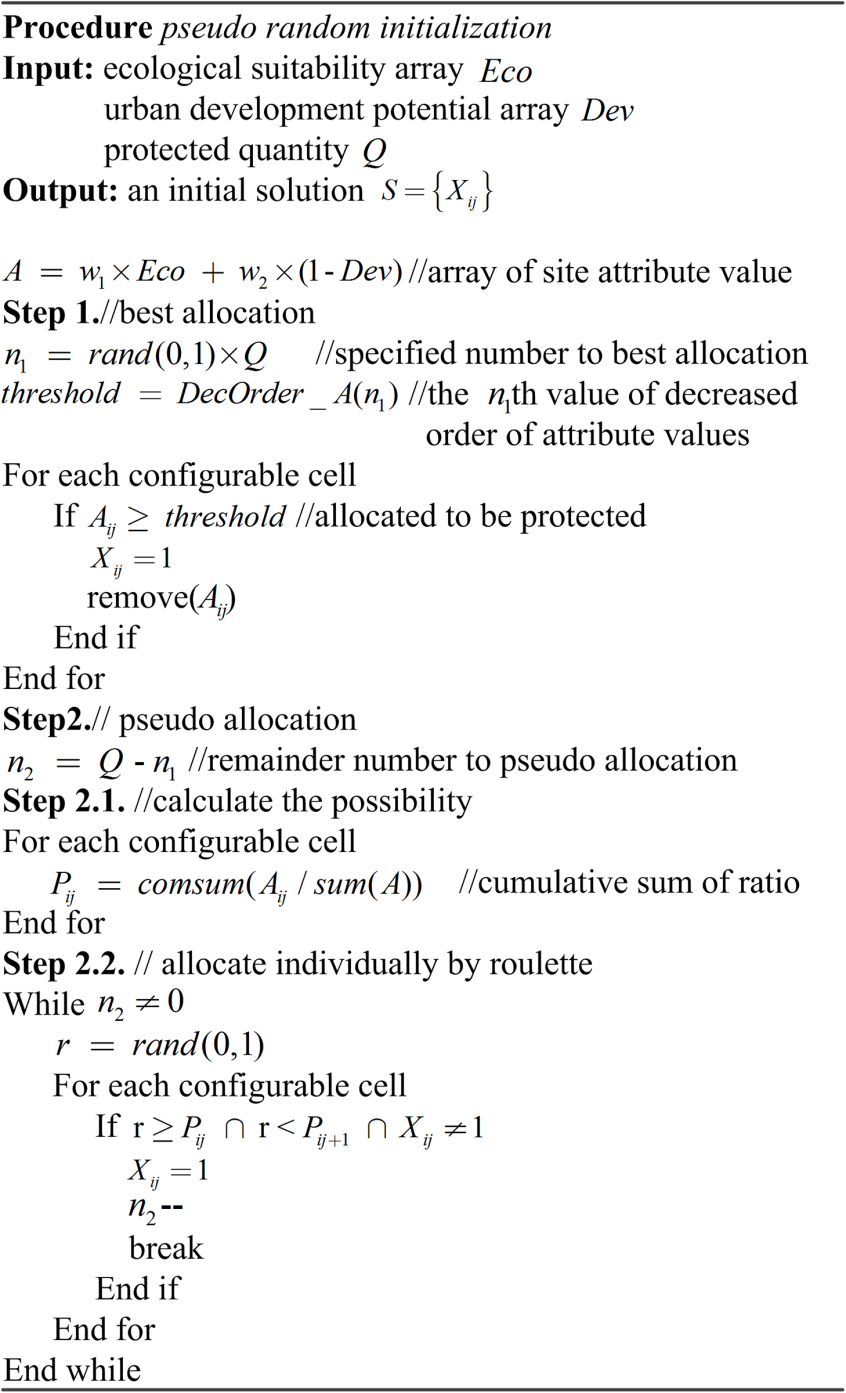
Pseudo code of the “pseudo-random” initialization.

The two initial methods are conducted according to a random proportion in the IABC algorithm. The pseudo code of the “pseudo-random” initialization method is shown in Fig 5.

### Neighbor solution generation strategy

In land allocation problems, operations of exchange, crossover, and mutation are widely used to generate neighbor solutions [45, 52]; thus, useful information can be shared among individuals. In the IABC algorithm, an exploitation search procedure is implemented for both employed bees and onlooker bees to generate neighbor solutions using the “replace” and “alter” operations, expressed in formula (7). Some region of the current solution u_j_ is replaced by the same region of its neighboru_k_. Meanwhile, to ensure the numerical balance between protected cells and non-protected cells, a reverse operation called “alter” is performed at a specified position outside the region for each replace operation.

$${\text{v}}_{\text{j}}={\text{u}}_{\text{j}}+\text{Replace}\left({\text{u}}_{\text{j}},{\text{u}}_{\text{k}}\right)+\text{Alter}\left({\text{u}}_{\text{j}}\right)$$ (7)

To generate a diversity of high quality neighbor solutions, two strategies for producing replacement areas and two strategies for the alter operation are adopted in this search procedure as follows:

- Replacement area 1 (R1): generate a rectangular region, with both the position and size of the rectangle set randomly, e.g., size = 3*2, located in (1,1), indicating row 1 and column 1.
- Replacement area 2 (R2): generate a multi-cell region formed by cells of poor quality, with the number of cells determined randomly, e.g., size = 0.1*Q, cells = {(1,2),….(8,90)}.
- Alteration strategy 1 (A1): choose the most suitable position to alter, as evaluated by the improvements in the objective function values in the neighborhood of the cell undergoing alteration.
- Alteration strategy 2 (A2): select a random position outside the replacement region, e.g., cell = (6,12).

**Fig 6:**
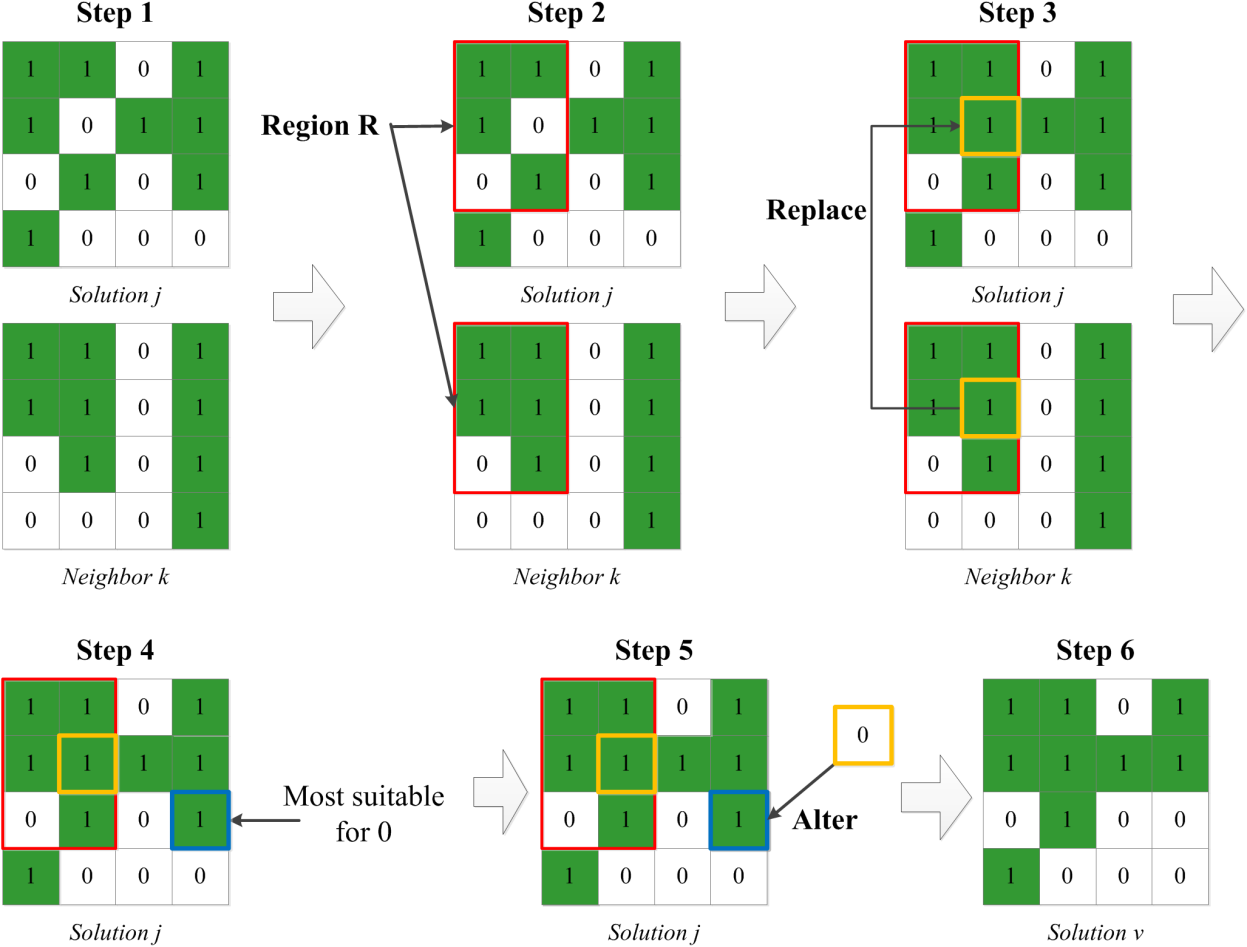
An example of the replace-and-alter strategy for generating neighbor solutions.

These strategies are selected randomly when executing the algorithm. Fig 6 gives an example of the combination of R1 and A1. In step 1, the solution j assigned by an employed or onlooker bee and a randomly chosen neighbor k are ready for the process. In step 2, a rectangular region R is generated randomly, and in step 3, the region R of solution j is replaced by the corresponding region of neighbor k. Because the code of the cell outlined in yellow is converted from 0 to 1 in region R, the amount of non-protected area is decreased while the amount of protected area is increased. To maintain the balance of protected and non-protected area, a reverse operation is performed outside region R. Among all of the outside cells that were already allocated to protected area, a position most suitable as a non-protected area (outlined in blue) was chosen in step 4 and encoded to 0 in step 5. In this way, a new feasible solution v, meeting the quantity constraint of the model, is generated in step 6.

### Local search strategy

**Fig 7:**
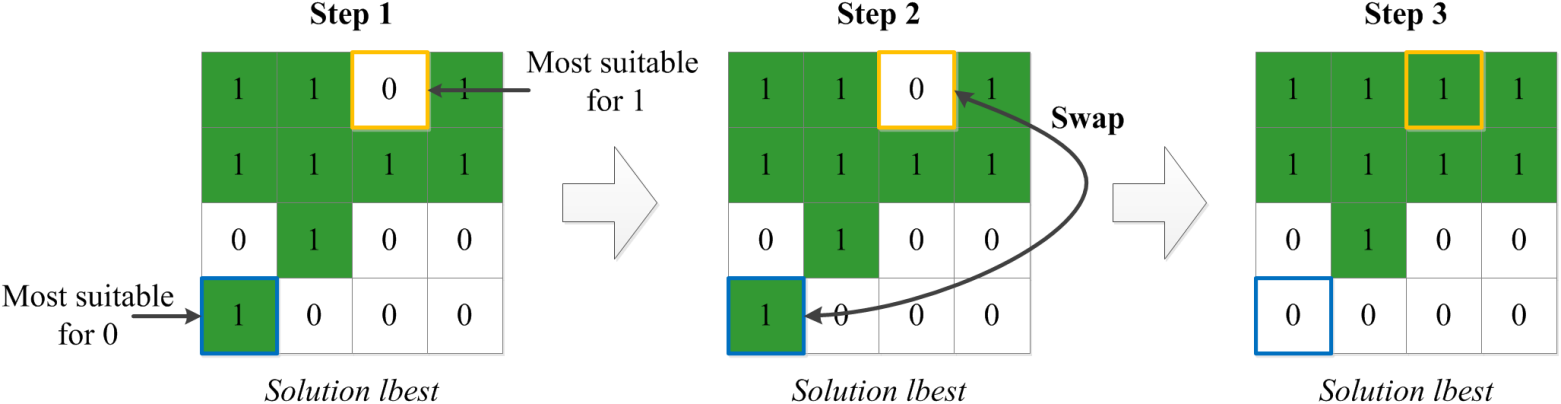
An example of the swap strategy for local search.

To enhance exploration ability and accelerate convergence speed, a local search strategy for the iterative optimal solution (known as local best solution, “lbset”), the “swap” operation, was embedded in the IABC algorithm. In the “swap” operation, the two most appropriate cells are exchanged. After changing the two cells to the new type of land, the objective function value in the neighborhood exhibits the largest improvement (detailed in Fig 7). In the local search procedure, the “lbest” solution experiences several “swap” operations, and the number of cell pair to exchange is set randomly within a predetermined range, e.g., range = (20,40) and number = rand(0,1)*20+20.

### Computational procedure

Due to the complexity of zoning the protected area, it is difficult to solve the problem effectively with a single strategy. The IABC algorithm fuses the initialization with multiple strategies and searches using “replace-and-alter” and “swap” operations. The algorithm stresses the balance of global exploration and local exploitation, and it also emphasizes the quality and diversity of the population during the searching process. The overall computational procedure is as follows:

Step 1: Set parameters “SN”, “Limit”, and “MCN”.
Step 2: Initialize population using two methods.
Step 3: If the “MCN” is reached, stop and output the global optimum; otherwise, perform Steps 4 through 10.
Step 4: For each employed bee, perform the following sub-steps:
  1. Generate a new neighbor solution by the “replace-and-alter” strategy and evaluate it.
  2. If the new solution is better, replace the old one with the new one.
Step 5: Calculate the probability values of the current solutions that are preferred by onlookers according to formula (6).
Step 6: For each onlooker, perform the following sub-steps:
  1. Select a food source to follow depending on the computed probabilities using a roulette-wheel mechanism.
  2. Use the same strategy to generate and retain a neighbor solution as an employed bee.
Step 7: Acquire the iterative optimal solution and improve it via the local search strategy.
Step 8: For each food source, if the “Limit” for abandonment is reached, send the scout to abandon the old solution and generate a new possible food source as the current solution.
Step 9: Store the global optimum.
Step 10: Go to Step 3.

## Experiments and analysis

The IABC program was implemented in MATLAB, and experiments in algorithm validation, application to Sanya region, and algorithm comparison were conducted. The experimental environment was an Intel(R) Core(TM) i5-3210M with a 2.50-GHz CPU and 4.00 GB RAM running Windows 7 OS.

### Algorithm validation

**Fig 8:**
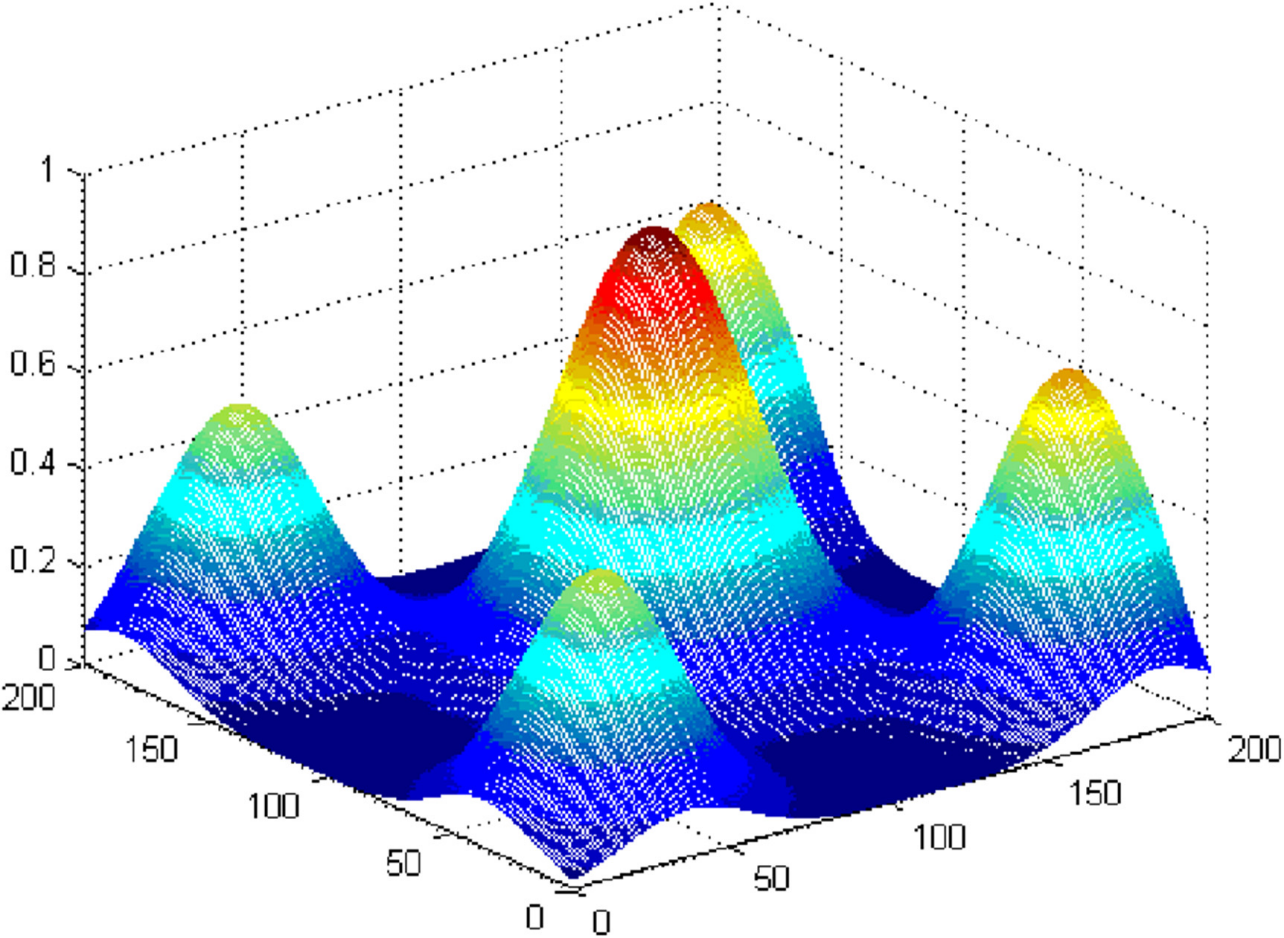
Simulated site attribute value surface with multiple peaks.

To verify the ability of the proposed IABC algorithm to escape a local optimum, a simulated data set with a known structure was optimized. Fig 8 shows the site attribute value surface with multiple peaks that was generated by Gauss functions. The highest values are located in the center, and there are four small peaks with lower values in the four corners. The data size was 200×200 cells, and the known global optimum is a circle with a radius of 20 cells located in the center. In this experiment, the protection number was set to 1,250, and the weights of the site objective and aggregation objective were set to 0.67 and 0.33, respectively.

**Fig 9:**
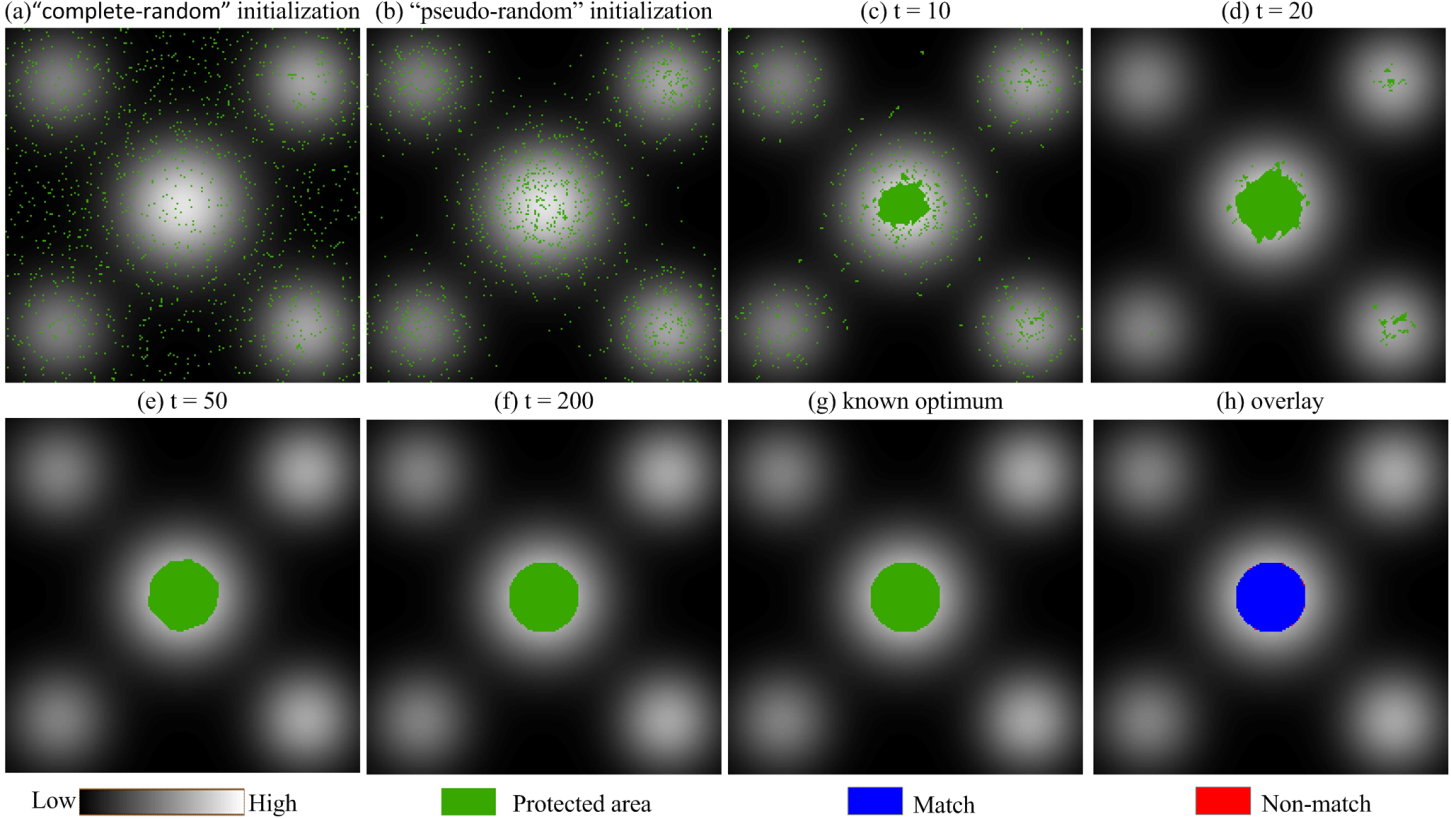
Optimization process and validation analysis of the IABC algorithm.

The optimization process and validation analysis of the algorithm are shown in Fig 9, whose base map is in black and white according to the site attribute values of the cells. In the initial stage, each solution in the primary bee population was generated in a “complete-random” or “pseudo-random” method, and the corresponding protected cells were distributed in the search space with different spatial characteristics; examples are shown in panels a and b. The solution generated by “complete-random” method was disorderly and unsystematic, whereas that generated by “pseudo-random” method tended towards the region with higher site attributes. In fact, regardless of how a solution was initialized, it was an essential component of the solution set, and the entire population worked together to carry the algorithm a step forward. As the search progressed, cells in the protected area slowly gathered, and those of the global optimum solution aggregated in the center and four corners, as shown in panels c and d. Then, the global optimum escaped the local optima in the four corners (panel e) through the communication of the artificial bees. After 200 iterations, the global optimal solution was fixed to the circle in the center, as demonstrated in panel f, and the optimization result was overlaid with the known global optimum (panel g). The vast majority of protected cells were completely matched (the blue cells in panel h), whereas only 0.8% of the cells were non-matched (the red cells in panel h) which might have been due to the discretizing of continuous data. Therefore, IABC was able to find the approximate optimal solution of the area optimization problem, and it exhibited an ability to escape local optima.

### Zoning protected ecological areas in Sanya

We applied the IABC to define an optimum scheme of protected ecological areas of Sanya. The “SN”, “Limit”, and “MCN” were set to 12, 150 and 1,000, respectively, and the weights of the three sub-objectives (w_1_, w_2_, w_3_, in formula (5)) were set to 0.34, 0.33, and 0.33, respectively. Equivalent weights were set for sub-objectives to test the methodology, and the following experiments and analysis only apply to the optimization under these given weights.

**Fig 10:**
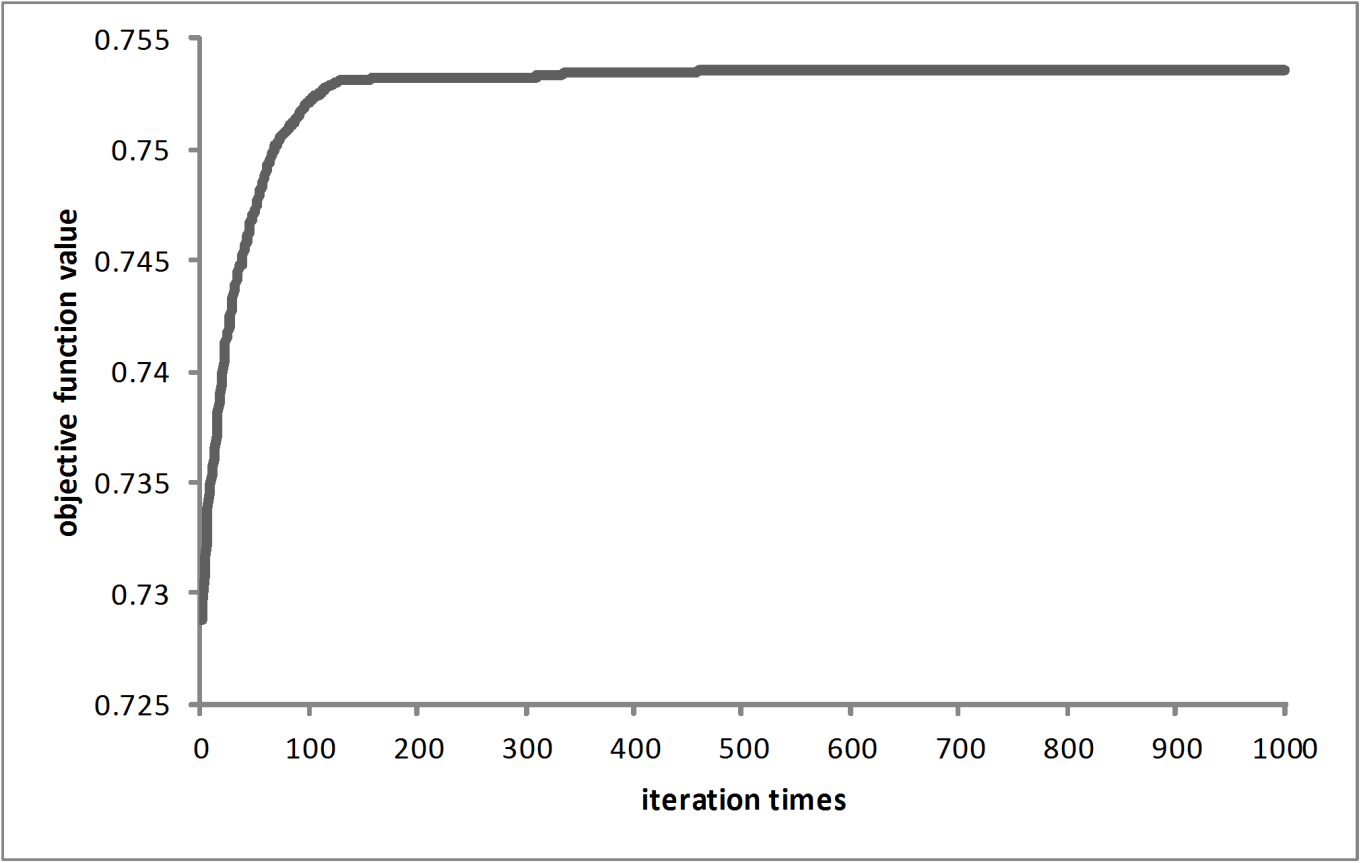
Improvement process of the global optimal objective function value for the IABC.

The improvement process of the global optimal objective function value for one run is shown in Fig 10. The objective function value was low at the initial stage but improved significantly as the optimization progressed. When the number of iterations was greater than 500, the value remained largely stable, improving only slightly in subsequent cycles.

**Fig 11:**
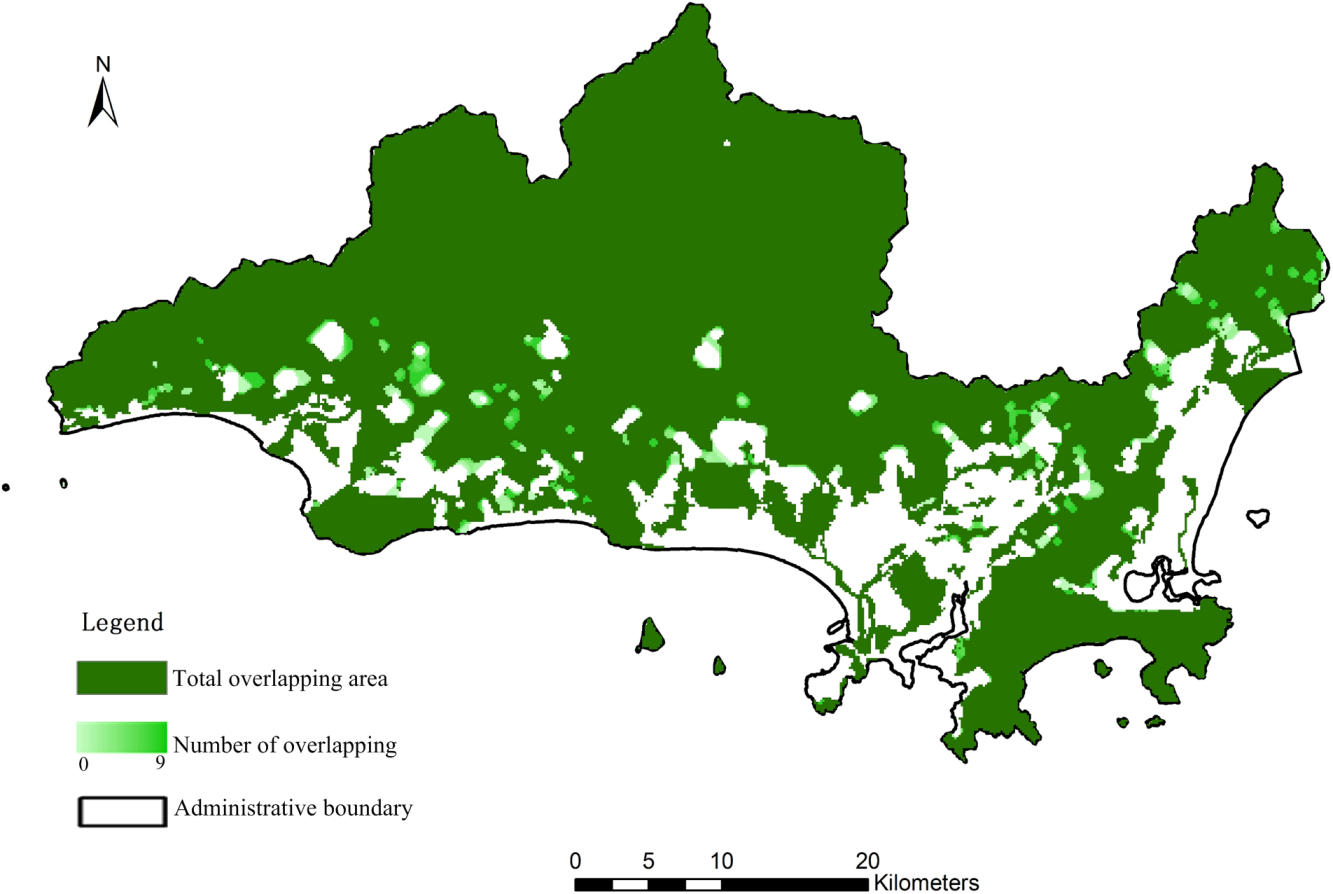
Overlay of the optimization results after repeatedly running the IABC algorithm 10 times.

Similar to other meta-heuristic algorithms, the optimization result might be influenced by randomness. Thus, we ran the IABC for ten repetitions, and Fig 11 shows the overlay of the ten optimization results. Although a few edges of the protected area did not completely overlap, the total overlapping area was 95.5% (the deep green areas). In the non-matched areas (shown as the gradient of light green), the number of areas overlapping more than 5 times accounted for the majority. Therefore, the IABC algorithm can generate stable solutions in the optimization process, demonstrating robustness.

**Fig 12:**
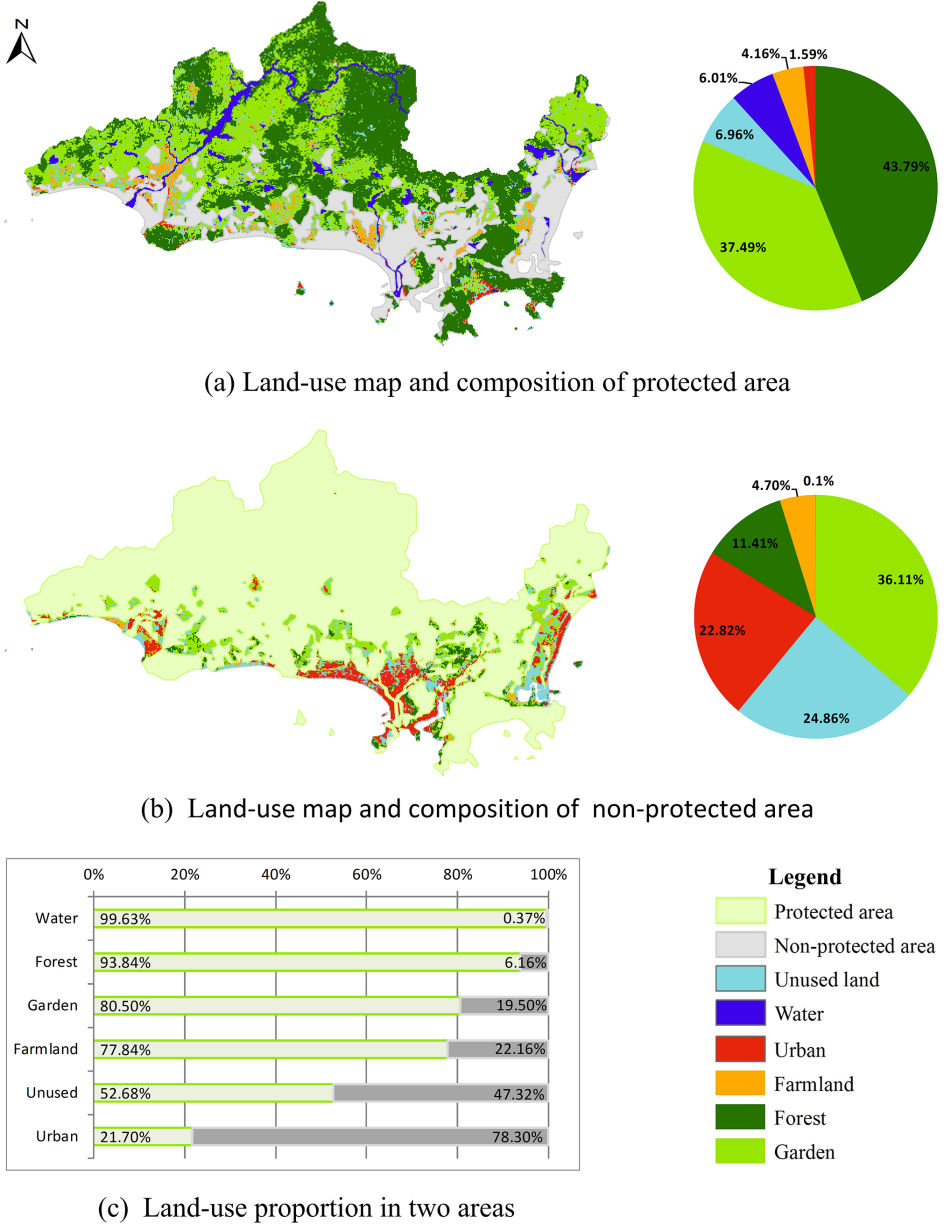
Overlay analysis of the IABC optimization result and land-use map.

To analyze the reasonableness of the planning result, an optimization result obtained by the IABC algorithm was overlaid with a land-use map as shown in Fig 12, and the quantity statistics result is provided in Table 3. The zoned protection area contained forest, garden, unused land, water, farmland, and urban area accounting for 43.79%, 37.49%, 6.96%, 6.01%, 4.16%, and 1.59% of the total protected amount, respectively (panel (a)). Thus, forest, garden, unused land, and water formed the main constituents of the protected area. The non-protected area was also covered by the six land-use types listed above, which accounted for 11.41%, 36.11%, 24.86%, 0.1%, 4.7%, and 22.82%, respectively, of the total non-protected area (panel (b)). As a result, garden, unused land, urban, and forest were the main components of the non-protected area.

**Table 3:** Land-use quantity statistics of the IABC optimization result.

Land-use	Regional amount	Protected amount	Non-protected amount
Forest	31435	29500	1934
Garden	31370	25252	6119
Urban	**4939**	1072	3867
Water	4061	4046	15
Farmland	3597	2800	797
Unused	**8903**	4690	4213
Total	84305	67360	**16945**

Each land-use type presented variable proportions in the two areas (panel (c)). Most of the water (99.63%), forest (93.84%), garden (80.5%), and farmland (77.84%) had been protected. A majority of urban area (78.3%) was clearly not protected. However, due to the gap between the total non-protected amount and the sum of the regional urban and unused land amounts (bold font in Table 3), it was impossible to cover the entire non-protected area with only urban and unused areas; moreover, some of these areas (21.7% and 52.68% for urban and unused areas, respectively) within the range of higher ecological benefit had been classified as protected area in interests balance. Consequently, some amount of farmland (22.16%), garden (19.5%), and forest (6.16%) near the urban region were configured as non-protected area and prepared for regional development, allowing for the susceptibility of such areas to human interference and their suitability for urban expansion, and in consideration of spatial compactness. The analysis result is affected by the accuracy of the remote sensing classification; and the specific percentage values may vary slightly; however the trend of allocation for each land use remains the same. Therefore, the optimal result can be recognized as an effective and reliable one.

As stated in the introduction, the aim of this paper is to propose a methodology that can generate an effective optimal result under given conditions and data. During the planning stage, several demand scenarios can be constructed through interaction and debate with stakeholders such as regional authorities, citizens, and nature-conservation agencies; and corresponding optimization schemes may be produced by changing the weights of the sub-objectives in the approach; thus, significant comparisons can be performed by policy makers. Although the weights can be changed, the ability to search for the optimal solution of the proposed algorithm is invariable.

### Algorithm comparison

To compare the performance of the IABC algorithm with other algorithms, three other methods were applied to zone the protected ecological area of Sanya using the same constraints and data: (1) AgentLA[15] (a free version is available at http://www.geosimulation.cn/AgentLA/), (2) ACO[5] (a free version is available at http://www.geosimulation.cn/), and (3) DS[23]. In addition, to evaluate the influence of the different initialization methods, a different version of improved artificial bee colony (IABC*) using the “complete random” method was introduced, and this initialization method was adopted by AgentLA and ACO.

**Fig 13:**
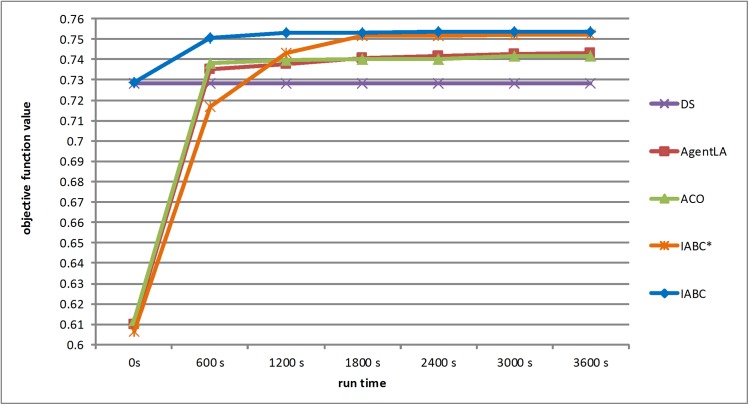
Curves of objective function value for the IABC, AgentLA, ACO, DS and IABC* methods (the IABC* method is the same as the IABC method except that initialization is implemented only by the “complete random” method).

Because the previous implementations of the AgentLA and ACO programs were encapsulated, it was difficult to acquire global optimal objective function values at every moment. Therefore, seven time points (0 s, 600 s, 1,200 s, 1,800 s, 2,400 s, 3,000 s, and 3,600 s) were selected as representative data for this study, and the current best objective function values obtained at each time point were compared among the different algorithms, as shown in Fig 13. The DS algorithm was performed in a single execution, and its objective value did not change over time. AgentLA and ACO performed similarly; their global optimal objective function values improved markedly in the beginning and continued to improve over the entire 3,600 s, but the rate of increase decreased over time. For the IABC* method, the initial values were very close to those of AgentLA and ACO, and its global optimal objective function value also improved substantially within 600 s, although not to the extent observed with AgentLA or ACO. After 600 s, IABC* maintained a high growth rate. Its global optimal objective function value started to exceed those of AgentLA and ACO after 1,200 s, and remained the advantage throughout the remaining time points. In addition, the IABC algorithm, which incorporated both the “complete random” and “pseudo-random” initialization methods, reached higher global optimal objective function values than the other methods early on, improving rapidly and then trending toward stability after 1,200 s. Thus, the IABC was able to find a superior optimal solution in the shortest time.

The IABC approach benefitted from the combined use of the two initialization methods. However, even when only the “complete random” method was used, the IABC* still revealed stronger searching ability and overmatched the AgentLA and ACO methods over 3,600 s. The curve nodes shown in Fig 13 indicate the global best solution in the entire population. Accordingly, although the series initial solutions were generated by the “complete random” method and the “pseudo-random” method, only the best solution among them is shown in the node 0 s of the IABC curve. Other solutions inferior to the best one participated in the iterations and were neighbors to supply useful information for the population. By employing various level initializations, the convergence speed of IABC would be greatly accelerated and the solution quality would increase slightly.

**Fig 14:**
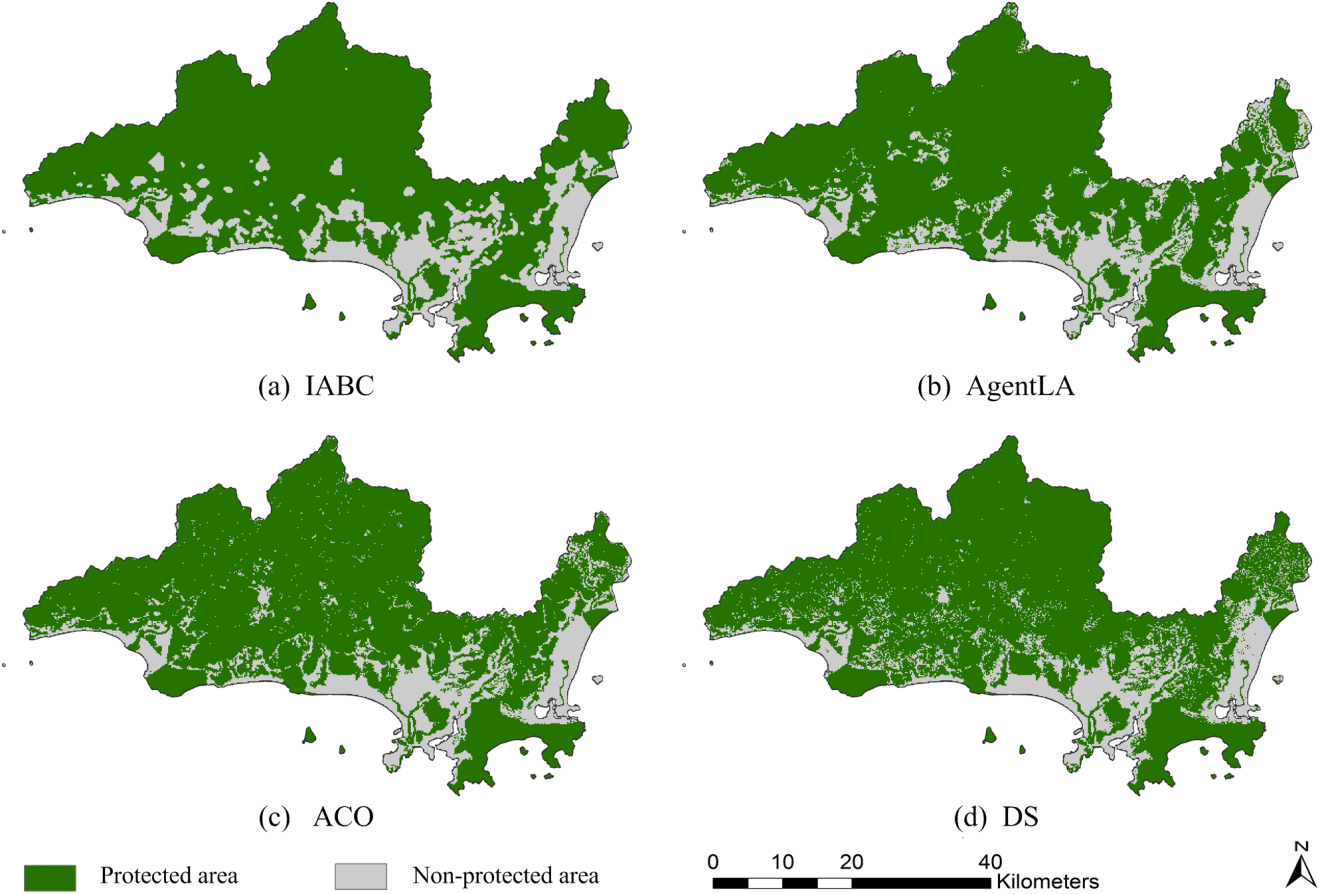
Examples of spatial patterns obtained by the (a) IABC, (b) AgentLA, (c) ACO, and (d) DS methods.

Ten executions of the IABC, AgentLA, and ACO methods were conducted, and the results of the statistical analysis are provided in Table 4. The maximum value obtained by the IABC was the highest of the methods, being 1.2%, 1.38%, and 3.36% higher than the maximum values of the AgentLA, ACO, and DS algorithms, respectively. In addition, the IABC had the lowest standard deviation, indicating a higher degree of stability. Fig 14 illustrates examples of the optimization results for the four algorithms at 3,600 s. Although simple and well understood, the spatial pattern obtained by the DS algorithm was the most fragmented (panel (d)). The results of AgentLA and ACO were relatively compact (panel (b) and (c)), whereas the IABC algorithm produced the most compact spatial pattern (panel (a)). Overall, the IABC algorithm effectively and efficiently generated a more optimal solution than did the AgentLA, ACO and DS methods.

**Table 4:** Statistical analysis of optimization results for ten executions of the IABC, AgentLA, ACO and DS methods.

Algorithm	Minimum value	Media value	Maximum value	Standard deviation	Gap
IABC	0.75324	0.75339	**0.75360**	**0.012%**	0.00%
AgentLA	0.74016	0.74278	0.74458	0.117%	1.20%
ACO	0.74031	0.74271	0.74320	0.079%	1.38%
DS	—	—	0.7283	—	3.36%

## Conclusions

The main contribution of this article is to introduce and improve the ABC algorithm for zoning protected ecological areas. There are three main improvements: a combination method to initialize the bee population, a “replace-and-alter” strategy to generate neighbor solutions, and a “swap” strategy for local search. Over a series of experiments, the IABC algorithm showed good convergence and robustness, and was able to escape local optima. It was also shown to be more effective and efficient than the AgentLA, ACO, and DS methods, and the optimal solution generated by the IABC algorithm was shown to be reasonable and reliable.

The quantitative model allows for the consideration of both site attributes and aggregation attributes. In addition, the proposed IABC method can serve as a reliable and efficient tool in the zoning procedure, enabling flexible and transparent comparison among different scenarios drawn by stakeholders. Our future work will incorporate more planning objectives to better express the willingness from stakeholders, such as GDP growth, the consistency to current land-use, and land conversion costs. Moreover, instead of obtaining a single alternative by the simple linear weighted method as presented in this article, a set of solutions called the Pareto front can be sought for multi-objective problems.

## Supporting Information

S1 Data The spatial variables for multi-criteria evaluations. (ZIP)
